# Assessment of stability and compatibility of dermatophagoides and blomia allergen mixture for sublingual immunotherapy

**DOI:** 10.1186/1939-4551-8-S1-A43

**Published:** 2015-04-08

**Authors:** Maytee Mateo Morejón, Damarys Toralba Averoff, Alexis Labrada, Anna Tsoraeva, Yunia Oliva Diaz, Mary Carmen Reyes Zamora, Raysa Cruz Jimenez, Beatriz Tamargo

**Affiliations:** 1 National Center of Bioproducts, Cuba; 2 Cuban Society of Immunology, Cuban Society of Allergy, Asthma and Clinical Immunology, Cuba; 3 University of Havana, Cuba

## Background

House Dust Mites are important etiological agents of respiratory allergy. Allergen mixtures are often used for specific immunotherapy in polysensitized patients, although scientific evidence to support its efficacy, safety and stability is scarce. Sublingual immunotherapy (SLIT) is being gradually accepted as an alternative to conventional subcutaneous immunotherapy. For SLIT, higher allergen doses are recommended, so the development of pharmaceutical formulations allowing the administration of such high doses is particularly relevant. Therefore, the aim of this work was to develop and evaluate liquid formulations of allergenic extracts of house dust mites: *Dermatophagoides pteronyssinus*, *Dermatophagoides siboney* and *Blomiatropicalis*, containing glycerol, for administration as sublingual vaccine.

## Methods

Starting from the freeze-dried active ingredients of standardized allergen products, developed by National Center of Bioproducts (BIOCEN, Cuba), there were obtained several formulation variants of the mixture of three allergens, with glycerol concentrations ranging from 20 to 50%. Biologic activity was measured by IgE inhibition ELISA and in-vivo Prick Test.

## Results

The mixture of three allergens, as well as, individual formulations at two concentration levels: 20 000 or 200 000 BU/mL, containing glycerol 20 or 50% showed stability at room temperature for 6 months in a similar extent, in contrast to aqueous formulation that at the highest concentration showed a fast degradation. Glycerol 20 or 50% was also effective as microbiological preservative agent.

## Conclusions

The technical feasibility and compatibility of ingredients of these formulations was experimentally demonstrated. The formulation containing 200 000 BU/mL, 10 times the concentration used for SCIT would be particularly suitable for SLIT.

